# Latent variable and clustering methods in intersectionality research: systematic review of methods applications

**DOI:** 10.1007/s00127-021-02195-6

**Published:** 2021-11-13

**Authors:** Greta R. Bauer, Mayuri Mahendran, Chantel Walwyn, Mostafa Shokoohi

**Affiliations:** 1 Epidemiology and Biostatistics, Schulich School of Medicine and Dentistry, Western University, London, ON Canada; 2 Social and Behavioural Health Sciences, Dalla Lana School of Public Health, University of Toronto, Toronto, ON Canada

**Keywords:** Systematic review, Intersectionality, Health equity, Research methods, Latent variable methods, Clustering methods

## Abstract

**Purpose:**

An intersectionality framework has been increasingly incorporated into quantitative study of health inequity, to incorporate social power in meaningful ways. Researchers have identified “person-centered” methods that cluster within-individual characteristics as appropriate to intersectionality. We aimed to review their use and match with theory.

**Methods:**

We conducted a multidisciplinary systematic review of English-language quantitative studies wherein authors explicitly stated an intersectional approach, and used clustering methods. We extracted study characteristics and applications of intersectionality.

**Results:**

782 studies with quantitative applications of intersectionality were identified, of which 16 were eligible: eight using latent class analysis, two latent profile analysis, and six clustering methods. Papers used cross-sectional data (100.0%) primarily had U.S. lead authors (68.8%) and were published within psychology, social sciences, and health journals. While 87.5% of papers defined intersectionality and 93.8% cited foundational authors, engagement with intersectionality method literature was more limited. Clustering variables were based on social identities/positions (e.g., gender), dimensions of identity (e.g., race centrality), or processes (e.g., stigma). Results most commonly included four classes/clusters (60.0%), which were frequently used in additional analyses. These described sociodemographic differences across classes/clusters, or used classes/clusters as an exposure variable to predict outcomes in regression analysis, structural equation modeling, mediation, or survival analysis. Author rationales for method choice included both theoretical/intersectional and statistical arguments.

**Conclusion:**

Latent variable and clustering methods were used in varied ways in intersectional approaches, and reflected differing matches between theory and methods. We highlight situations in which these methods may be advantageous, and missed opportunities for additional uses.

## Introduction

As a framework for incorporating social power, heterogeneity, and community relevance into health research, intersectionality has gained prominence as an analytic approach to qualitative and more recently quantitative methods [1]. Emerging from U.S. Black feminist communities [2, 3] and into Black feminist academic work in legal studies and sociology [4–6], intersectionality focuses on social power structures and the ways oppression is configured at intersections of race, gender, class, and other axes of marginalization [6]. Importantly, it centers the experiences of those at the social margins in an embodied way [7, 8], with the central recognition that experiences and well-being are shaped by mutually constituted social identities or positions. This is to say that, for example, the experiences of Black lesbians cannot be understood by adding together the average effects of being Black, being a sexual minority, and being a woman [7]. As such, as intersectionality has been taken up within quantitative research, researchers have sought out methods that allow variables to act together in ways that may better reflect intersectional analytic approaches. One such set of methods involves grouping individuals based on how multiple variables cluster together, producing new categories reflecting meaningful patterns of multiple attributes. Such methods fall into the broad categories of latent variable methods and clustering methods.

Health researchers have highlighted latent variable methods such as latent class analysis (LCA) and latent profile analysis (LPA) as data-driven analytical approaches for examining intersectional stigma [9]—a concept that represents the influence of combined and overlapping oppressions to form a distinct positionality [10]. These analytic methods take a “person-centered” approach by which subgroups of individuals are identified based on their stigma experiences, rather than a “variable-centered” approach in which individuals are not grouped and stigma is assumed to impact everyone similarly [11]. Latent variable methods are model-based approaches; they assume an underlying statistical model for the population from which the data were obtained and identify unobservable (latent) groups within that population [12]. LCA uses a set of observed *categorical* variables to identify latent classes [13], wherein individuals are homogenous within classes (i.e., with high probability of similar response patterns regarding the measured variables), while heterogeneous across classes [11]. LCA assumes conditional independence, that is, the measured variables are conditionally independent given latent class membership [13]. Given the type of observed data, multiple extensions exist, including LPA which similarly creates classes/profiles but using *continuous* variables, and latent transition models (LTA) used for longitudinal data [14]. As model-based methods, goodness-of-fit statistics such as the Akaike information criterion [15], Bayesian information criterion [16], sample size adjusted BIC [17], and entropy [18] are available to aid in decision-making about the number and features of the latent groups. Fit is one consideration in addition to parsimony, model stability, and interpretability [13]. Details regarding LCA model building, selection, interpretation, and presentation of results have been described elsewhere [19, 20].

The second broad class of methods includes non-model-based clustering methods such as hierarchical cluster analysis (HCA) and non-hierarchical cluster analysis (e.g., K-means clustering) [21]. Like latent variable methods, they allow for classification based on clustering on a range of measured variables. Unlike latent variable models that intend to recover unobservable observations in the data (using multiple observed variables), their main goal is only data-driven identification of clusters, and goodness-of-fit statistics are not available [20, 22]. Agglomerative HCA, for example, considers each individual as a single cluster; then, most-similar clusters are grouped together in an iterative process until bigger clusters, all mutually exclusive, are created [21, 22]. The similarities or dissimilarities of the identified clusters are taken into account using *distance* measures (e.g., Euclidean distance algorithm) or *similarity* measures, followed by cluster validation [22]. Also, multiple cluster agglomeration (or linkage) methods have been proposed for measuring dissimilarities between two clusters, for example, nearest neighbor, centroid clustering, and Ward’s method [22]. Unlike latent variable methods, clustering methods typically accommodate multiple variable types within one analysis, as values are transformed using standardization techniques. Clustering results are typically depicted in a tree-like dendrogram.

The relationship between intersectionality and latent variable or clustering methods requires elucidation. Core tenets of intersectionality suggest different potential uses for such person-centered approaches. For example, the most foundational tenet that social categories are not independent, but interlocking [4, 23, 24] may suggest creation of clusters/classes using social identities or positions such as gender, ethnoracial group, and education. Another tenet is that these identities/positions are linked to social and structure inequity [23], which may suggest creation of clusters/classes related to social-structural processes such as sexism, racism, or classism. However, similar approaches based in other frameworks (e.g., social determinants of health) have been used without labeling them intersectional [25]. In addition, generation of new categorical measures produced using these types of methods may be driven by pragmatic rather than theoretical objectives, for example to reduce variable numbers. Moreover, classes or clusters are often not themselves the ultimate goal of research studies. Rather, these new categorical variables are used within additional analyses in varying ways. Ultimate goals may be descriptive or analytic, either describing inequalities in health or other outcomes across classes/clusters, or using these new classifications in analytic studies of processes that drive inequalities [26].

In this review, we sought to evaluate uses of latent variable and clustering methods across disciplines, in academic articles that explicitly stated an intersectional approach. We aimed to describe study characteristics, engagement with intersectionality, and methods used, and to evaluate match between intersectionality and methods to provide guidance for future health equity research.

## Methods

### Search strategy

The systematic review was conducted according to the Preferred Reporting Items for Systematic Reviews and Meta-Analyses (PRISMA) guidelines [27], though the standard assessment of the risk of bias in included studies was not deemed relevant as our interest was characterizing methods rather than evaluating evidence from study results. As a part of a larger intersectionality methods review [1], researchers conducted a systematic search of all quantitative English-language research articles from 1989 (the year the word was introduced in academic literature [5]), through May 12, 2020, wherein the authors explicitly stated they were applying an intersectionality theoretical framework. A search of Scopus (including Medline) and ProQuest Political Science and Public Administration (including PsycINFO) identified papers with titles, abstracts or keywords containing “intersectional*”. Titles and keywords containing “qualitative” were excluded from the search [1].

### Selection strategy

Papers were de-duplicated using Covidence Systematic Review Software [28]. To determine eligibility, papers were jointly title and abstract screened by two independent reviewers, and full-text articles were screened by a single reviewer. Interrater reliability during screening was 92.5%, and conflicting decisions were resolved via consensus-based discussions. Papers were deemed eligible for inclusion into the study if they were peer-reviewed original quantitative or mixed methods studies or methods papers that explicitly stated the use of an intersectional framework. Measure development or validation papers were excluded, as while clustering methods play a role in scale validation it is for a very specific purpose that is not usually intersectional in nature. Finally, all papers that did not use latent variable or clustering analyses in an explicitly intersectional way were excluded. In total, 16 papers were identified as eligible for inclusion.

### Data extraction

Data items were extracted into an Excel sheet, which had been pilot tested and revised to improve clarity. Data on latent variable or cluster methods were extracted by two independent reviewers (MM and CW), with data checks conducted to validate extraction results. The following data items were extracted: (a) *Article characteristics* (journal discipline, country of first author’s home institution, research approach, study design, sampling method, sample size, use of a health outcome), (b) *Incorporation of the intersectionality framework* (explicitly defining intersectionality, citing foundational authors, citing key methodological papers), (c) *Latent variable and clustering methods* (type of method used, rationale for method used, variables used in method, number of groups created, approach used to choose final number of groups, group variable names), and (d) *Use of group variable in additional analyses* (type of statistical method conducted using the group variable, role of group variable in analysis, ability for the results to vary by intersection, reporting results for all intersections). Journal discipline was categorized by grouping initial classifications from within Ulrich’s Serials Analysis System [1, 29]. Citation of foundational intersectional authors was recorded as any citation of the Combahee River Collective [2], legal scholar Kimberlé Crenshaw [4, 5], or sociologist Patricia Hill Collins [6]. Number of key methodological intersectionality papers cited was coded based on 45 papers identified in our earlier large interdisciplinary systematic review of quantitative intersectional methods in papers published from 1989 to 2020 [1]. Finally, quotes were extracted wherein authors discussed their rationale for their methods choices.

### Data analysis

Measures of frequency were estimated for each extracted data item using SAS version 9.4.1. [30]. Extracted author quotes were reviewed to identify elements of authors’ rationales for their latent variable or clustering methods use.

## Results

**Fig. 1:**
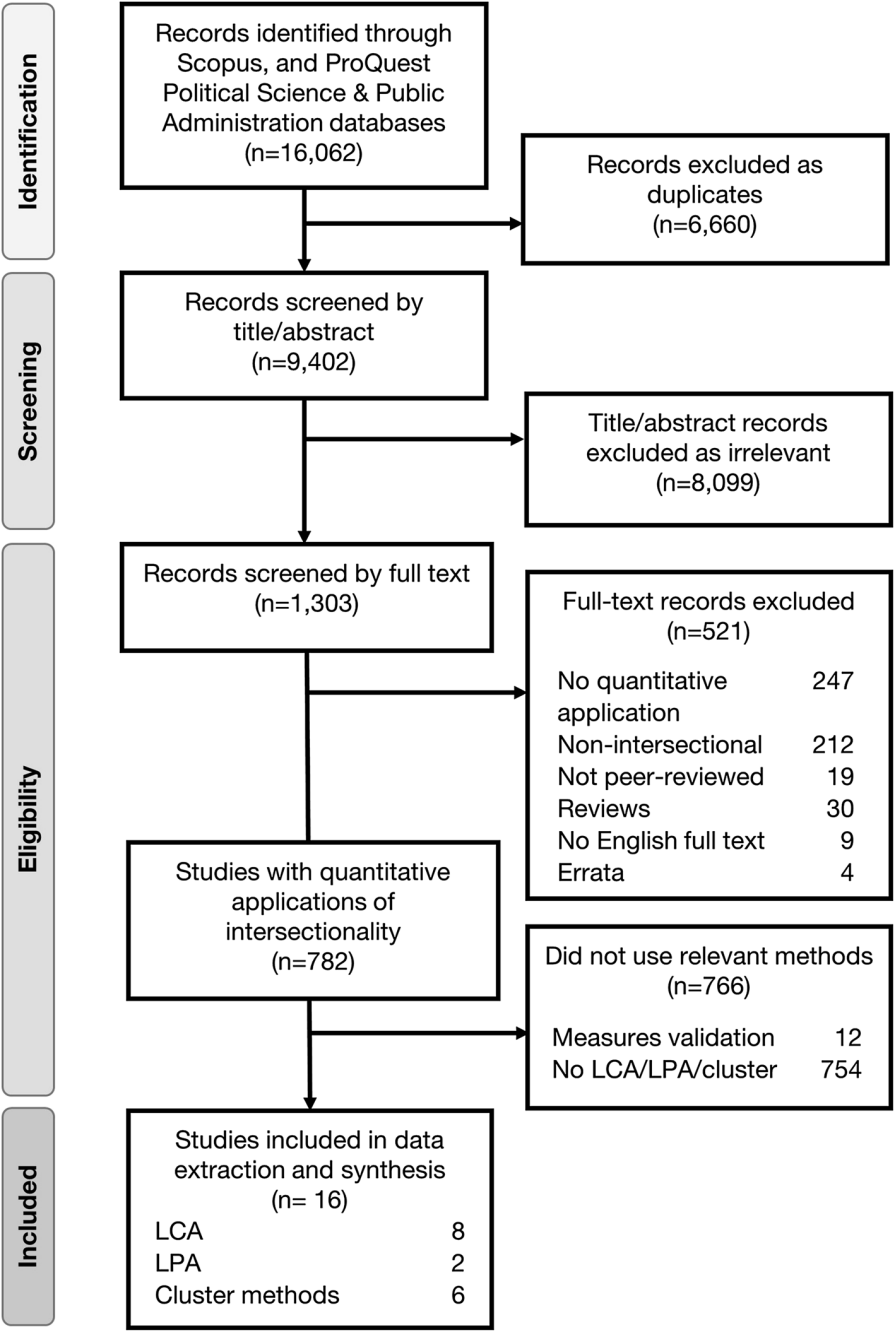
Flow diagram of systematic review process and exclusion criteria. *LCA *latent class analysis; *LPA* latent position analysis

Following full-text review, 782 quantitative intersectionality application papers remained, of which 16 (2.0%) used a latent variable or clustering method and were included in extraction and analysis for this paper (Fig. 1). Table 1 summarizes the characteristics of included papers and indicators of their engagement with intersectionality. Of the 16 papers, 10 (62.5%) included a health-related outcome, while some others focused on areas such as education that may be considered social determinants of health. Papers were primarily in psychology, social sciences, and health disciplines, had U.S. lead authors, were quantitative only and cross-sectional, and traversed the full range of sample sizes. Fourteen of 16 papers (87.5%) defined intersectionality, and 15 (93.8%) cited foundational authors. Engagement with methodology papers varied, with 4 (25.0%) citing none of the 45 methods papers and 7 (43.8%) citing just one.

**Table 1:** Characteristics and indicators of engagement with intersectionality in quantitative intersectionality papers using latent variable or clustering analysis

	Total (*n* = 16)	
	*n*	%
Health-related outcome	10	62.5
Journal discipline^a^		
Psychology	9	56.3
Other social sciences^b^	4	25.0
Children and youth	4	25.0
Medical and life science	3	18.8
Sociology	2	12.5
Population/public health and safety	2	12.5
Education	2	12.5
Other sciences^c^	1	6.3
Country of first author		
United States	11	68.8
United Kingdom	4	25.0
Germany	1	6.3
Study type		
Quantitative	14	87.5
Mixed-methods	2	12.5
Study design		
Cross-sectional study	16	100.0
Complex multi-stage sample	4	25.0
Data from census or population records (e.g., birth records)	0	0.0
Intersectionality defined	14	87.5
Cited foundational author(s)	15	93.8
Engagement with methodology papers^a^		
0 cited	4	25.0
1 cited	7	43.8
2–4 cited	4	25.0
5 + cited	1	6.3

^a^Multiple disciplines per journal; proportions do not sum to 100%

^b^Other than psychology, sociology, criminology

^c^Other than medical and life sciences, or physical, earth or space sciences

^d^Based on the list of 45 methodology papers included in a larger review [1]

Details on applications of intersectionality in these papers are summarized in Table 2. Of the 16 papers, 8 used latent class analysis, 2 used latent profile analysis, and 6 used clustering methods. The 16 papers included a total of 15 models (in 14 papers) for which clear clusters/classes were presented. Of these, 9 (60.0%) included 4 classes/clusters; 3 included fewer and 3 included more. For all but one paper, all variables used to form the classes/clusters were based in social power. This included social position variables, like age or gender, processes such as discrimination, and identity-related measures like race centrality. The remaining study created profiles based on diagnoses and medication usage, with subsequent analyses using the resulting profiles stratified by intersections of ethnicity/race and gender.

**Table 2:** Quantitative papers explicitly applying latent variable or clustering analysis methods within an intersectionality framework

Method used	Paper	Sample	Variables used in LCA/LPA/clustering	Clusters	Analyses conducted with resulting cluster categories
Latent class analysis (LCA)	Garnett, et al., 2014 [39]	965 high school students	8 dichotomous variables. Past 12 months discrimination because of (yes/no for each): Race, ethnicity or color; Self or family from another country; Someone thought you were gay, lesbian, or bisexual; Weight. Past 12 months bullied or assaulted because of (yes/no for each): Race, ethnicity or color; Self or family from another country; Someone thought you were gay, lesbian, or bisexual; Weight.	1: Low discrimination 2: Racial discrimination 3: Sexual orientation discrimination 4: Racial and weight discrimination with high bullying (intersectional class)	Multinomial logistic regression with sociodemographic characteristics as exposure, and latent class membership as outcome. Linear and logistic regression models with latent classes as exposures, controlling for sociodemographics, to assess three mental health outcomes.
	Bécares and Priest, 2015 [45]	10,115 eighth-grade students	42 dichotomous variables, with the following each measured at 3–5 time points from kindergarten to 8th grade. Individual-level variables: Child lived in a single-parent household; Household below the poverty threshold level; Food insecurity; Parental expectations of child’s future academic achievement; Whether parents had moved since previous interview; Household-level composite index of socioeconomic status. School-level variables: % students eligible for free school meals; % students from a racial/ethnic background other than white non-Hispanic. Neighborhood variables: Neighborhood safety level	1: Individually and contextually disadvantaged 2: Individually wealthy, contextually disadvantaged 3: Individually and contextually wealthy 4: Individually disadvantaged, contextually wealthy	Looked at differences in each of the eight academic and non-academic outcomes between cross-classified race/ethnicity*gender*SES groups, where SES was defined by latent class membership.
	Budge et al., 2016 [35]	442 trans-identified individuals	1 dichotomous and 4 categorical variables. Dichotomous: Race—person of color. Categorical: Gender (4 categories); Sexual orientation (8 categories); Education (5 categories); Household income (8 categories).	1: Socioeconomic and racial privilege 2: Educational privilege	T-tests with latent classes as exposures, to assess differences in anxiety and depression.
	Byrd and Carter Andrews, 2016 [36]	1468 middle and high school students	22 dichotomous variables. Source of discrimination (yes/no for each): Peers; Teachers; Administrators; Front office personnel; counselors/social workers; Others; No discrimination. Perceived reason for discrimination (yes/no for each): Race/ethnicity; Gender; Religion; Social class/family’s financial status; Sexual orientation; Disability; Other attribution. Form of discrimination (yes/no for each): Don’t get called on in class by the teacher; Experience name calling; Fewer opportunities to access people in the school that can help me succeed; Excluded from certain social groups; Excluded from academic opportunities; Excluded from social opportunities; Punished more than other students; Other form.	1: Adult cluster 2: Multiple cluster 3: Peer cluster 4: Low cluster	Chi-square tests to assess differences in sociodemographic characteristics among latent class memberships. One-way ANOVA and Tukey post-hoc tests with latent class membership as exposure, to assess differences in discrimination frequency and school-related experiences and perceptions.
	Landale et al., 2017 [31]	1789 Latino and non-Latino white adults	6 dichotomous and 1 categorical variable. Dichotomous: Latino ethnicity; Age—30 years or older; Education—high school or higher; Speaks English; Employment status; Below poverty threshold. Categorical: Immigration status (3 categories).	1: U.S. born and advantaged whites 2: U.S. born and advantaged Latinos 3: U.S. born, young, jobless Latinos 4: Documented and older Latinos 5: Undocumented and disadvantaged Latinos	Linear and logistic multilevel models with latent class membership as the exposure (individual-level), and controlling for neighborhood- and individual-level covariates, for three discrimination-related outcomes.
	Goodwin et al., 2018 [37]	1052 community members	3 dichotomous and 7 categorical variables. Model 1 (only SES variables): Categorical: Social occupational class (4 categories); Employment status (4 categories); Household income (3 categories); Housing tenure (4 categories); Education (3 categories). Dichotomous: Receiving benefits (excluding pension or child benefits); Past year non-mortgage related debt; Moved twice or more in past 2 years. Model 2 (includes Model 1 variables plus the following): Categorical: Immigration status (4 categories); Race/ethnicity (6 categories).	Model 1: 1: Professional, homeowners 2: Professional, renters 3: Skilled, renters 4: Students, renters 5: Economically inactive, renters 6: Economically inactive, homeowners Model 2: 1: Professional, homeowners, White British 2: Economically inactive, renters, White British 3: Students, mixed tenure, non-migrant, mixed ethnicity 4: Skilled, renters, non-migrant, mixed ethnicity 5: Economically inactive, homeowners, mixed migration status, mixed ethnicity 6: Professional, renters, migrant, mixed ethnicity 7: Economically inactive, renters, migrant, mixed ethnicity	For both model 1 and model 2, logistic regression models with latent class membership as exposure, adjusting for age and gender, for the outcome “common mental disorder”.
	Earnshaw et al., 2018 [40]	1293 adults living in low-resource communities	9 dichotomous variables. Discrimination (ever experienced, never experienced). Discrimination attributions (binary ever/never for each): Race/ethnicity; Income; Age; Gender; Appearance; Language; Weight; Sexual orientation.	1: Single attribution 2: No discrimination 3: Several attributions 4: Most attributions	ANOVA and chi-square analyses with latent classes as exposure, assessing differences in sociodemographics, discrimination experiences, and health outcomes.
	Gazard et al., 2018 [32]^a^	1052 community members	Same variables as Model 2 from Goodwin et. al., 2018 [37]	1: Migrant, mixed ethnicity, low SES 2: White British, low SES 3: Non migrant, mixed ethnicity, student 4: Non migrant, mixed ethnicity, skilled 5: Mixed migration status, mixed ethnicity, economically inactive 6: Migrant, mixed ethnicity, high SES 7: White British, high SES	Chi-square tests to look at differences in discrimination experiences, mental/physical disorder and long standing illnesses between the latent classes. Logistic regression models with health service use as the outcome, and latent class membership as the exposure. Logistic regression models with health service use as the outcome, and discrimination as the exposure. Unadjusted and adjusted models were presented. Here the latent classes were used in the adjusted models, alongside other variables.
Latent profile analysis (LPA)	Shramko et al., 2018 [44]	219 Latinx sexual minority youth	6 continuous variables. Scales: Perceived bias-based victimization due to sexual orientation or gender identity; Perceived bias-based victimization due to Latinx identity; Perceived discrimination due to sexual orientation or gender identity; Perceived discrimination due to Latinx identity; Ethnic centrality; Sexual orientation centrality.	1: Low perceived discrimination and victimization and low identity centrality 2: Low perceived discrimination and victimization, but high identity centrality 3: Moderate perceived discrimination and victimization, and moderate centrality 4: High perceived discrimination and victimization, but moderate identity centrality	ANOVAs and chi-square tests to assess differences in demographic characteristics between latent profiles. Structural equation modeling looking at the association between the profiles and the following outcomes: GPA, depressive symptoms, self-esteem.
	Taggart et al., 2019 [38]	1170 African-American and Caribbean Black adolescents	7 continuous variables. Religiosity (4 subscales): Organizational participation; Religious support; Nonorganizational participation; Subjective religiosity. Racial identity (3 subscales): Racial centrality; Racial public regard; Racial private regard.	1: Low intersected identity 2: High intersected identity 3: High racial identity 4: High religiosity	Chi-square analyses to determine differences in class membership by demographic variables. Cox proportional hazards survival analysis to model the association between profile membership and sexual initiation.
Cluster analysis	Stirratt et al., 2008 [51]	40 lesbian, gay or bisexual adults	Each participant was asked to select up to 12 identities that best represented them. Had to include at least gender, racial/ethnic group, and sexual identities. Then for each identity selected, rated how it applied to a set of 70 attributes (binary variable for each of the 70).	N/A (cluster analysis conducted for each participant)	Separate cluster analysis for each respondent. Then used results/structure of each respondent’s cluster analysis to create identity measures. Then: 1. Correlated identity measures with other measures (psychological well-being, social well-being, collective self-esteem, internalized homophobia, depression). 2. Chi-square and ANOVA analyses to assess differences in identity measures between race*gender subgroups.
	Aspinall and Song, 2013 [33]	326 mixed-race individuals	16 dichotomous variables. Salient identities based on (yes/no for each): Age or life-stage; Kind of study or work; Level of education; Level of income; Political belief; Family; Ethnic group or cultural background; Country family came from originally; Regional identity; Nationality; Religion; Skin color; Social class; Gender; Disability; Sexuality/sexual orientation.	N/A, clusters not named (cluster analysis presented visually on dendrogram)	Description of how attributes were clustered together based on the dendrogram visualization. No further analyses conducted.
	Brown et al., 2018 [41]	116 African-American female college students	Model 1: 2 continuous variables: Racial socialization; Gender socialization. Model 2: 2 continuous variables: Ethnic socialization; Gender socialization.	Model 1 (racial-gender clusters): 1: Racial silence and low gender tradition cluster 2: High racial influence and high gender tradition 3: Minimal racial coping and low gender tradition Model 2 (ethnic-gender clusters): 1: High ethnic influence and high gender tradition 2: Minimal ethnic pride and minimal gender tradition 3: High ethnic influence and low gender tradition 4: Ethnic silence and low gender tradition	For each cluster set, MANCOVA analyses used to examine if identity cluster profiles were associated with the outcomes of sexual assertiveness and safer sex behaviors. Mediation analysis used to examine mediating role of sexual assertiveness on the association between the ethnic-gender clusters and the outcome of safer sex behavior.
	Whaley and Dubose, 2008 [34]	322 undergraduate students	16 dichotomous variables. Ever received mental health care or counseling for (yes/no for each): Mood disorder; Anxiety disorder; Substance-related disorder; Eating disorder; Adjustment disorder; Impulse control disorder; Stereotypic movement disorder. Ever used medications to treat the previous conditions. Currently receiving mental health care or counseling for (yes/no for each): Mood disorder; Anxiety disorder; Substance-related disorder; Eating disorder; Adjustment disorder; Impulse control disorder; Stereotypic movement disorder. Currently using medications to treat the previous conditions.	1: History of psychiatric treatment for emotional disorders 2: Addictive behaviors to cope with depression 3: Loss of control over eating behavior 4: Pharmacotherapy for clinical depression	For four ethnicity/race*gender intersectional groups, separate regression analyses conducted to assess the effect of the four profiles on the outcome cumulative frequency percentages of psychological problems.
	Price et al., 2019 [43]	946 high school students	2 dichotomous and 1 categorical variable. Dichotomous: Race—youth of color; Sexual orientation—LGBQ. Categorical: Sex and gender (3 categories).	1: LGBTQ youth 2: Heterosexual youth of color 3: Heterosexual white youth	ANOVA and logistic regression to assess differences in school and well-being related outcomes across clusters. ANOVA and chi-square tests to assess differences in discrimination and bullying across racial and gender.subgroups within LGBTQ cluster. Regression mediation analysis to examine the mediating role of discrimination on the association between cluster membership and the outcomes—well-being, depression and grade point average.
	Wanka et al., 2019 [42]	400 Turkish adults	4 continuous variables. Neighborhood discrimination due to: Ethnic origin; Age; Religion; Gender.	1: Cluster 1 2: Cluster 2 3: Cluster 3 4: Cluster 4	Descriptive statistics regarding how clusters differed by social-spatial variables. ANOVA to analyze differences between the clusters with regard to neighborhood satisfaction and sense of home. Linear regression models used to examine the association between cluster membership and the outcomes neighborhood satisfaction and sense of home.

^a^Uses model 2 LCA results from Goodwin et. al., 2018 [37], to assess different outcome in the same sample

Across studies, the resulting classes/clusters were then frequently used as a variable in additional analyses. Analyses generally either described differences in sociodemographic characteristics between the classes/clusters, or used class/cluster membership as exposure groups in analyses such as regression analysis, structural equation modeling, mediation, and survival analysis, to predict outcomes. These methods typically resulted in coefficient estimates and statistical inferences regarding the significance of class/cluster membership towards the outcome. Some studies in addition to using clusters as terms in their statistical models, presented overall mean or prevalence estimates for each cluster [31, 32], but not all did. One study performing a cluster analysis presented results visually in a dendrogram, with no specific definition of the final clusters, and no assessment of the clusters relative to any outcome or sociodemographic [33].

Author quotes regarding their methods rationale included both theoretical/intersectional and statistical arguments. Theoretical reasons included the importance of a “person-centered approach”, to examine the complexity of co-occurring experiences, to identify intersectional subgroups or response patterns, to find intersectional constructs such as intersectional stigma or discrimination, and appropriateness to researchers’ relative power in data analysis. One study using multidimensional scaling analysis describing the method as a way of applying an “idiographic” approach, which is an approach that reveals underlying or hidden constructs [34]. Author rationales are summarized in Table 3, and illustrated with sample quotes.

**Table 3:** Theoretical rationale provided by authors for use of latent variable or clustering methods within an intersectionality framework

Rationale	Exemplar quotes
Person-centered approach	“The intersectionality framework proposes that individual experiences must be examined in the context of simultaneous memberships in different status groups. In emphasizing constellations of multiple statuses, this framework requires a person-oriented approach instead of a variables-oriented approach” [31]
Examining the complexity of co-occurring experiences	“Discrimination and bullying are complex social phenomena … As opposed to attempting to disentangle the different attributes of discrimination and bullying, LCA can examine how they jointly co-occur” [39] “LCA can create a series of classes that allows for the study of not only multiple disadvantaged positions, but also those positions of privilege, as well as positions that occupy both” [37]
Identifying intersectional subgroups or response patterns	“With trans populations often treated as one homogeneous group, it is unclear how levels of depression and anxiety may differ across trans subgroups or for individuals holding other minority identities. Therefore, we used cluster analysis to identify patterns across social identities”. [35] “In this investigation, we were attempting to determine which patterns (i.e. the quality) of racial-gender and ethnic-gender socialization were reported by participants.” [41]
Finding intersectionality	“We first employed latent class analysis to understand who reports intersectional experiences of discrimination.” [40] “Idiographic and idiothetic approaches using MDS [multidimensional scaling analyses] provided evidence of intersectionality for African-American college students.” [34]
Appropriate to researchers’ relative power and privilege	“[C]luster analysis … is appropriate given the lack of existing empirical evidence to test more explicit associations of study constructs. … helps us to minimize the assumptions we may make as researchers with relative power and privilege in absence of guiding theory and empirical support for testing more specific associations between study constructs.” [35]

Statistical arguments sometimes focused on the advantage of methods such as LCA over non-clustering methods. LCA was seen as useful in that it was a data-driven exploratory approach [35], and that it did not require a priori specification of groups [36]. The groups were noted to be potentially both more homogenous [37] and substantively meaningful [35, 38], especially useful when groups are then used as categorizations to predict outcomes. For example, one study stated, “Using an LCA approach allowed us to define more cohesive social groups and subsequently the reference group in the regression analyses was also likely to be a more homogenous group, which increases the validity of the analyses.” [37]. Some authors provided a rationale for choosing one type of latent variable or clustering method over others. Cited advantages of LCA over other clustering methods included the availability of model fit statistics to aid in decision-making regarding class number [35, 36]. In practice, all LCA/LPA studies in our review used fit statistics, and some also considered theory or interpretability of the final classes [31, 32, 37–40]. Additionally, certain cluster studies utilized statistical criteria as well, either alone [30, 38])) or in combination with interpretability [43]. Other stated advantages of LCA compared to cluster methods included the model-based nature of the method allowing for groupings to be tested in independent samples to confirm generalizability [36], and analyses allowing for bootstrapping estimations [35].

In using LCA to combine multiple indicators of socioeconomic status, it was noted to better show nuanced differences than methods that combine socioeconomic indicators into a single continuous variable (e.g., principal components analysis) [37]. In contrast, authors using hierarchical clustering or other non-LCA/LPA methods cited advantages such as not assuming independence of observed variables, and the ability to accommodate both continuous and categorical variables [43].

## Discussion

Included studies were those published in English. Given intersectionality’s roots in the United States, relatively few studies have been published in other languages, but those are not represented here. While intersectionality approaches have been used in quantitative research across a wide range of disciplines [1], latent variable and clustering methods in the current study were used primarily in psychology, social sciences, and health research. This reflects the roots of latent variable methods in mental health research, and disciplinary culture regarding methods. In comparison with our larger systematic review [1], authors using these methods seemed more engaged with intersectionality, as evidenced by the provision of definitions (87.5% vs. 73.1% in the larger literature), citation of foundational sources (93.8% vs. 68.0%) and citation of any of a list of key intersectionality methods papers (75.0% vs. 53.0%). Below, we highlight considerations regarding the match between applications of latent variable and clustering methods and intersectional tenets and research aims, address some limitations of current approaches, and highlight missed opportunities for additional uses of these methods.

### “Person-centered” approaches and relationality

Authors commonly described both latent variable and clustering methods as person-centered rather than variable-centered methods [31, 36, 38, 43–45]. This distinction emerged in personality psychology in the 1970s as a way to understand individuals based on multiple personality-related variables that were seen to pattern in specific ways within individuals [46]. In research applications, person-centered approaches came to be understood as a search for groups of individuals who were homogenous with regard to whether and how particular variables affected others [11, 47, 48]. In epidemiological terms, person-centeredness would then appear to be about using multiple measures to identify meaningful categories of individuals across which there is causal effect modification for associations under study. However, classes/clusters were not used in this way in any of the included studies, and understandings of person-centeredness in published papers were broader or unarticulated.

It is easy to see how the language of person-centeredness may be appealing to researchers taking an intersectional approach, in that it may be seen as mapping onto the core intersectionality concept of relationality. Relationality is the idea that phenomena such as race/ethnicity, sex/gender and social class are interconnected and maintained through relational processes [24]. Thus, health at an intersection, for example, cannot be understood as the sum of its social identity parts [7]. A relevant aspect of relationality here is co-formation, in which old categories may not be useful and new co-formed ones may be needed [24]. While not labeled clearly as such, this concept appeared to be what some authors referenced when they wrote about identifying an “intersectional class” [39] or finding “evidence of intersectionality” [34]. As intersectionality is more commonly understood in research as an analytic approach or paradigm than a finding [23, 49], it may be more appropriate to be specific regarding findings, for example describing finding evidence to support co-formation of gender and race rather than finding intersectionality itself.

### Intersectional stigma versus intersections of social identities or positions

While an occasional study brought social intersections into the analysis after clustering (e.g., analyzed effects of intersection on outcome within strata of mental health clusters [34]), most analyses conceptualized intersectionality as primarily represented within the classes/clusters. These approaches comprised two groups based on objectives for implementing latent variable or clustering methods, the first creating intersectional classes/clusters reflecting stigma or social processes, and the second reflecting classes/clusters of social identities/positions.

Many papers in the first group of studies sought to create classes/clusters based on stigma, discrimination, or victimization in ways that traversed different social positions (e.g., by combining measures such as homophobia and racism) [36, 39, 40, 42, 44]. This use harkens back to Berger’s foundational work on intersectional stigma, wherein she notes it is composed of influences that can be tied to specific social positions, but which in totality create qualitatively different forms of intersectional stigma [10]. Other authors took a process-based approach focused on socialization, community involvement, or identity centrality, rather than discrimination [38, 41]. There were two general approaches to these studies of intersectional processes. In the first, researchers analyzed processes within a particular intersection, such as African-American women [41] or sexual minority Latinx youth [44]. In the second, they aimed to create process-based classes in general samples, such as students [39] or residents of a low-resource geographic area [40]. The first approach seems to clearly map onto an intracategorical approach to intersectional complexity, which focuses on specific experiences within an intersection [50], while the second may capture intercategorical categories of stigma or other social processes.

A second group of studies sought to create classes based on social identities or positions themselves, using three main approaches. The first was to create a set of classes/clusters based on a standard set of social positions, such as race, gender, income, and immigration status [32, 35, 37]. The second was to use these methods to create a single measure of a multidimensional construct, for example socioeconomic status [37, 45]; these classes could then be combined with other social positions to form intersections. The classes/clusters created were then typically used as a variable in additional analyses. Another identity application focused specifically on within-person interaction of identities, rather than their relationship with other variables. This involved more unconventional approaches, such as performing a separate cluster analysis for each person [51] or assessing the correlation between identities for each person in a matrix, then using that matrix data to do the cluster analysis [33].

While the creation of classes/clusters from a standard set of social identities/positions to create “intersections” was common in the included studies, the limitations of this application were generally left unacknowledged with regard to both statistical assumptions and to applicability and interpretability of results to those living at different social intersections. Statistically, the assumption of conditional independence in LCA would require that latent class membership explain all the shared variance between included social identities/positions (e.g., income, race, trans identity, sexual orientation, and education [35]). This could require that entrenched disparities (e.g., racial disparities in income), be fully explained by latent “intersections”, a substantial assumption. We note that Bayesian LCA may be useful in detecting and addressing violations of conditional independence [14]. With regard to interpretability, we acknowledge the appeal of using cluster methods in this way to incorporate multiple social position variables into analyses with smaller sample sizes. For example, it is more feasible to condense six binary social position variables into one four-category variable, rather than conduct an analysis of 64 (2^6^) intersectional categories. However, presenting class/cluster results as “intersections” may mask important heterogeneity and limit interpretability. Classes/clusters based on social identities/positions are not homogenous in membership. LCA classes are based in conditional probabilities, and the resulting classes may not correspond to identifiable groups for health-related interventions. This suggests a trade-off between simplified data and potential applicability to real-world intersections.

### Data-driven versus theory-driven approaches

The tension between theory-driven and data-driven methods in data analysis was clear in the papers’ discussions of advantages and disadvantages. Data-driven approaches do allow for condensing a large number of variables in the absence of a theoretical basis for classification. Some researchers stated that the more data-driven nature of these methods serves to mitigate researchers’ relative power by reducing the impact of researchers’ assumptions [35]. Nevertheless, theory and subjectivity were still required both in the choice of variables used and in the naming and interpretation of resulting classes/clusters. Moreover, researchers often do have information on which to base a choice of intersection for study, and a wealth of qualitative and quantitative research, and community knowledge, to draw from in making these decisions. Moreover, deliberate incorporation of community knowledge can be structured into research team membership and processes, as well as study design, addressing issues of social power that are central to intersectionality [23, 52]. As such, while particular steps in the approaches used may be described as data-driven, in truth all papers we included drew on theory as well. We note that if there is a strong argument for data-driven analytic approaches, it would also apply to other methods (e.g., decision trees [1]) that also serve to take a data-driven approach to reducing intersectional categories in high-dimensional data [53]. Unlike latent variable or cluster methods, decision tree methods result in clearly defined categories by identity/position.

### Advantages and further opportunities for latent variable and clustering methods

The ability of latent variable and clustering methods to allow for overlapping and co-occurring experiences or identities/positions (and to statistically accommodate correlated variables) allows this set of methods to play a unique role in moving complex theory into quantitative analysis. Under what conditions are these methods most advantageous? While latent variable or clustering methods can result in categories that are similar to those that would have been logically coded, they also have the potential to identify multidimensional constructs that may represent qualitatively different categories of experience (e.g., individual and contextual wealth and disadvantage throughout childhood [45]). This reflects the original aspirations of person-centeredness. These meaningful new categories can then be incorporated into more conventional analyses.

Combining continuous variables is also a strength of latent position and clustering methods. While a series of categorical variables may be cross-coded into categories (potentially representing intersections), cross-classifying continuous variable would first require categorizing each variable. Given that cut-points are often arbitrary and categorization results in loss of information, latent profile analysis or clustering have an advantage in retaining full information, and can be used to create a categorical profile variable that may be more meaningful.

Across the reviewed papers, we noted potential uses of latent variable and clustering methods that were not observed, but which may provide opportunities to contribute to intersectional understandings of health. These missed opportunities included uses in longitudinal analysis, multilevel analysis, effect-measure modification, mediation analysis, and mixed methods. All papers reviewed used cross-sectional analyses, though one coded classes based on multiple retrospective time points. Because co-formation of intersectional experience occurs in social context and over the life course, there is an opportunity to expand analysis over longitudinal periods. While latent variable and clustering methods involve combining measures at a point in time, the resulting classes/clusters represent new categorical variables that could then be used in conjunction with follow-up data to study how class/cluster membership is associated with later outcomes. Moreover, opportunities to examine how membership in classes itself changes over time (e.g., latent transition analysis, longitudinal LCA) could be taken up in future work.

Quantitative intersectionality researchers have called for greater use of multilevel data to reflect social context and structural factors [54, 55]. As “person-centered methods” the methods studied were unsurprisingly all applied at the level of the individual. It is worth noting that person-centered methods consider the person in a developmental context and as an “integrated psychological, biological and social being” who must be understood in the context of their environment [48]. While the methods in our review were not designed for nested data, it would also be possible, for example, to conduct an LCA analysis at the group level. We found two attempts to incorporate contextual factors in analyses within this review. One study used factors from three social-ecological levels—the individual, school, and neighborhood—in their LCA analysis, though these were applied to individual-level observations rather than in nested data to create a measure of advantage/disadvantage for each participant [45]. Another study used individual-level variables in an LCA analysis but then used the resulting classes as an individual-level variable within a multilevel regression [31].

In our review, intersectional classes/clusters were frequently treated as exposure variables in subsequent analyses. We did not note any use as effect-measure modifiers. Given the original conceptualization of person-centered methods as producing homogeneous classes with regard to the effects of exposures on outcomes, and the fit of this conceptualization with intersectionality perspectives that acknowledge differential impacts across intersections, we would recommend further exploration of uses of classes/clusters as effect-measure modifiers or stratification variables. The potential roles of intersectional stigma classes, for example, could plausibly modify the effects of a range of exposures on health. Such an approach could also be embedded in a mediation analysis that accommodates interaction between cross-coded intersections as an exposure and a variable such as intersectional stigma as a mediator. Intersectional mediation approaches allow for decomposition of the indirect effect into components representing effects of different mediating classes, versus class membership having different effects on the outcome for those at different intersections (mediated interaction) [26, 56]. Finally, we note that while our review was of quantitative methods, two papers reviewed included mixed methods, which may have additional advantages with regard to an intersectional approach.

## Conclusion

Researchers applied latent variable or clustering methods in a range of ways that reflected different matches between intersectionality and their data analysis methods. While limitations were noted, there appears to be underdeveloped potential in applying these and related clustering methods to questions of intersectional co-formation, particularly with regard to experiences of stigma, discrimination, and violence, or in identifying and encoding multidimensional constructs representing complex social positioning. The use of these classes/clusters in further analyses would benefit from greater complexity, including use in longitudinal and multilevel studies, and in studies of effect-measure modification. Finally, research would benefit from greater specificity in reporting of rationale for methods and interpretation of findings, to better support the ultimate goal of improving health equity.
